# Diabetic cardiomyopathy: Early diagnostic biomarkers, pathogenetic mechanisms, and therapeutic interventions

**DOI:** 10.1038/s41420-023-01553-4

**Published:** 2023-07-21

**Authors:** Jin-Ling Huo, Qi Feng, Shaokang Pan, Wen-Jia Fu, Zhangsuo Liu, Zhenzhen Liu

**Affiliations:** 1 Traditional Chinese Medicine Integrated Department of Nephrology, The First Affiliated Hospital of Zhengzhou University, Zhengzhou, 450052 P. R. China; 2 Research Institute of Nephrology, Zhengzhou University, Zhengzhou, 450052 P. R. China; 3 Henan Province Research Center For Kidney Disease, Zhengzhou, 450052 P. R. China; 4 Key Laboratory of Precision Diagnosis and Treatment for Chronic Kidney Disease in Henan Province, Zhengzhou, 450052 P. R. China; 5 Department of Chinese Medicine, The First Affiliated Hospital of Zhengzhou University, Zhengzhou, 450052 P. R. China

**Keywords:** Cardiomyopathies, Drug development

## Abstract

Diabetic cardiomyopathy (DCM) mainly refers to myocardial metabolic dysfunction caused by high glucose, and hyperglycemia is an independent risk factor for cardiac function in the absence of coronary atherosclerosis and hypertension. DCM, which is a severe complication of diabetes, has become the leading cause of heart failure in diabetic patients. The initial symptoms are inconspicuous, and patients gradually exhibit left ventricular dysfunction and eventually develop total heart failure, which brings a great challenge to the early diagnosis of DCM. To date, the underlying pathological mechanisms of DCM are complicated and have not been fully elucidated. Although there are therapeutic strategies available for DCM, the treatment is mainly focused on controlling blood glucose and blood lipids, and there is a lack of effective drugs targeting myocardial injury. Thus, a large percentage of patients with DCM inevitably develop heart failure. Given the neglected initial symptoms, the intricate cellular and molecular mechanisms, and the lack of available drugs, it is necessary to explore early diagnostic biomarkers, further understand the signaling pathways involved in the pathogenesis of DCM, summarize the current therapeutic strategies, and develop new targeted interventions.

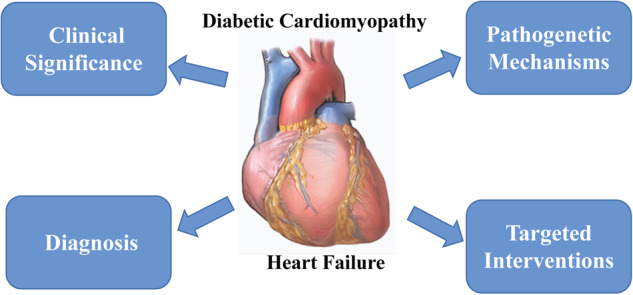

## Facts

- Cardiomyocyte apoptosis plays a key role in the pathogenesis of diabetic cardiomyopathy.
- Targeting hyperglycemia reduces cytotoxicity in cardiomyocytes.
- Some new and old small-molecule chemical drugs are in ongoing clinical trials for the treatment of DCM.
- Traditional Chinese medicine and some natural products can exert protective effects against diabetic cardiomyopathy.

## Open questions

- How do novel biomarkers identify early diabetic cardiomyopathy?
- What is the mechanism by which hyperglycemia leads to cardiomyocyte apoptosis?
- How is hyperglycemia involved in the progression of cardiac fibroblast activation?
- Can some emerging treatment strategies for diabetic cardiomyopathy be used in clinical practice?

## Introduction

With the growth of the economy, changes in lifestyles and diets, and the aging of the population, the number of diabetic patients and disease incidence are increasing at an alarming rate. According to statistics from the International Diabetes Federation, as of 2021, there were ~537 million people aged 20–79 with diabetes worldwide, and it is expected that this number will reach 784 million by 2045. Diabetic cardiomyopathy (DCM), which was first introduced by Rullber in 1972, is a common and severe complication of diabetes that can lead to the development of heart failure. DCM, which is the dominant cause of heart failure in patients with diabetes, is caused by abnormal glucose metabolism, resulting in structural heart defects and dysfunction without other cardiac risk factors, such as coronary artery disease, hypertension, and severe valvular diseases [1]. At present, although there are therapeutic strategies available to treat DCM, treatment is mainly focused on controlling blood glucose and blood lipids, and there is a lack of effective drugs or strategies targeting damaged myocardial tissue. Therefore, understanding the clinical symptoms, identifying early and highly sensitive diagnostic biomarkers, elucidating the underlying pathogenetic mechanisms, and developing new targeted interventions for DCM, are critical for improving the prognosis of patients and preventing the occurrence and development of the disease.

## Clinical symptoms and diagnosis of DCM

DCM was initially described as a diabetes mellitus (DM)-induced pathophysiological condition in which cardiac dysfunction and heart failure occurred in the absence of coronary artery disease, hypertension, and other valvular heart disease. The initial symptoms of DCM are mild left ventricular stiffness, slightly decreased compliance, and diastolic dysfunction, which are easily ignored by patients, thus missing the best opportunity for diagnosis and treatment. The subsequent clinical manifestations of DCM are arrhythmia, angina pectoris, and eventually the development of congestive heart failure, which is life-threatening in some severe cases. At present, many noninvasive techniques, including electrocardiography, echocardiography, chest radiography, pulsed-wave Doppler tissue imaging, computed tomography, magnetic resonance imaging, and endocardial biopsy, have been used to detect changes in cardiac architecture and function [2]. Endocardial biopsy, which is the gold standard for the clinical diagnosis of DCM, can detect cardiac hypertrophy, necrosis, myocardial fibrosis, and other pathological changes, but this method cannot show the abnormal diastolic function of the heart in the early subclinical stage and is associated with a certain degree of trauma [3]. Given the rare clinical symptoms in the early stage of DCM, routine noninvasive examinations are almost ineffective. Therefore, researchers have focused on investigating feasible methods for the early diagnosis of DCM.

Evidence has shown that the serum glycosylated hemoglobin (HbA1c) and cardiac troponin I (cTnI) levels in the DCM group were significantly higher than those in the diabetes mellitus alone group, suggesting that serum HbA1c and cTnI levels were closely related to DCM and could serve as promising diagnostic markers for DCM [4, 5]. Some studies have proposed that the increase in atrial natriuretic peptide (ANP), brain natriuretic peptide (BNP), and O-GlcNAc can be used as biomarkers of DCM [6–8], as well as the increase in calcium-sensitive receptors in cardiomyocytes and restoration of calcium homeostasis in type II DCM [9]. NT-proBNP is significantly increased in DCM and can be used to determine cardiac function in diabetes [10, 11]. The detection of serum markers, such as increased inflammatory mediators (TNFα, isoprostanes, IL-6, and C-reactive protein), elevated fibrotic markers (TGFβ1 and IGFBP7) and decreased antioxidant markers (leptin, adiponectin, and bilirubin), can also provide a basis for the early diagnosis and treatment of DCM [12]. Recent studies have shown that a number of new novel markers, such as galactin-3 (Gal-3), adiponectin (APN), and irisin, are significantly changed in the clinical course of the various stages of DCM [13]. Moreover, evidence suggests that exosomes, microRNAs, and long noncoding RNAs have potential applications as biomarkers for the detection of DCM [14, 15]. A clinical trial of novel biomarkers for the diagnosis and treatment of DCM has been in progress (ChiCTR1900027080). The novel study of the prediction and evaluation of DCM in patients with type 2 diabetes mellitus by magnetocardiogram vectors has been in registered and is in clinical phase 1 (ChiCTR2100049400). Exploring new promising biomarkers that can identify early DCM is critical for reducing the mortality of patients with DCM.

## Pathogenetic mechanisms of DCM

**Fig. 1:**
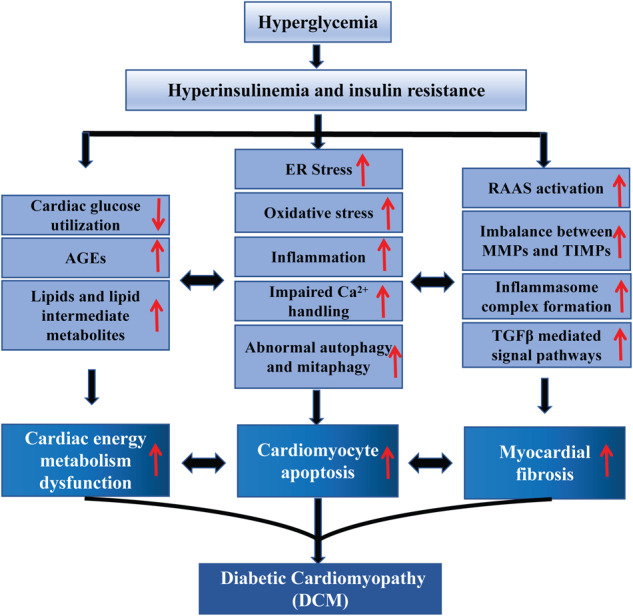
Pathogenetic mechanisms of DCM. Persistent hyperglycemia induces hyperinsulinemia and insulin resistance and then causes abnormal glucose metabolism, cardiomyocyte apoptosis, and myocardial fibrosis. Lipids and lipid intermediate metabolites, advanced glycation end products (AGEs), endoplasmic reticulum stress (ERS), oxidative stress, inflammation, impaired Ca^2+^ handling, autophagy, mitophagy, renin–angiotensin–aldosterone system (RAAS) activation, imbalance between MMPs and TIMPs and TGFβ-mediated signaling pathways are all involved in the progression of DCM.

The pathogenetic mechanisms of DCM are intricate and are still not fully understood. Chronic hyperglycemia exerts toxic effects on the myocardium during the progression of DCM through direct and indirect pathways, inducing cardiac remodeling, diastolic and systolic dysfunction, and eventually severe heart failure. In this review, we summarized the reported pathogenetic mechanisms of DCM (Fig. 1).

### Abnormal glucose metabolism and insulin resistance

**Fig. 2:**
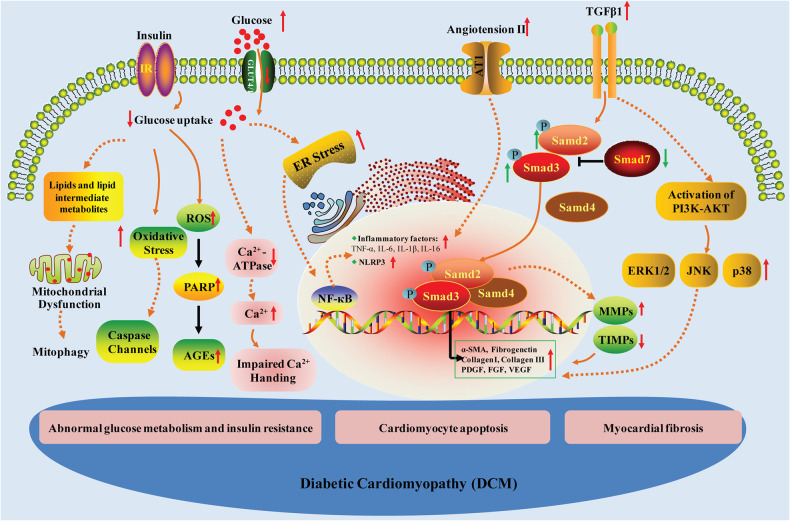
Key pathogenetic mechanisms involved in DCM progression.

Persistent hyperglycemia, which is the initiating factor of DCM, reduces glucose clearance and increases gluconeogenesis. Under normal physiological conditions, glycolysis provides only 30% to 40% of the cardiomyocyte energy demands to sustain systolic and diastolic function [2]. The uptake of glucose depends on glucose transporters 1 and 4 (GLUT1 and GLUT4). DCM causes a decrease in GLUT1 and GLUT4 in myocardial cells, leading to decreased glucose uptake and abnormal glucose metabolism [16]. Moreover, glucotoxicity in DCM patients increases the formation of advanced glycation end products (AGEs) in myocardial cells, which cross-link with extracellular matrix (ECM) proteins, resulting in increased cardiac fibrosis and impaired myocardial diastole (Fig. 2) [17].

Glucose utilization is significantly limited by hyperinsulinism and cardiac insulin resistance in DCM patients [18]. Due to impaired glucose utilization, almost all myocardial energy metabolism comes from the oxidation of nonesterified fatty acids, resulting in the deposition of lipids and lipid intermediate metabolites, such as ceramide, diacylglycerol, and uncoupling protein-3 in cardiomyocytes. The accumulation of these substances affects the energy supply of the myocardium, causing cardiomyocyte necrosis, myocardial fibrosis, and myocardial dysfunction and eventually contributing to abnormal cardiac structure and function [17]. In addition, numerous studies have confirmed that chronic hyperglycemia promotes the excessive release of reactive oxygen species (ROS) through the electron transport chain. ROS activate poly ADP-ribose polymerase (PARP), directly mediate glycosylation and inhibit phosphoglyceraldehyde dehydrogenase, blocking the normal glycolytic pathway and inducing other harmful cascade reactions, including an increase in AGEs and the activation of protein kinase C (PKC) [19, 20]. AGEs can stimulate collagen expression and accumulation, promote the cross-linking of collagen, and lead to increased myocardial fibrosis and decreased cardiac muscle compliance [17, 21, 22]. Under high glucose conditions, the activity of glucose transporter-4 is decreased, which reduces the transmembrane transport of glucose into cardiomyocytes, reduces the uptake of glucose by cardiomyocytes, and then affects the energy metabolism of cardiomyocytes, leading to diabetic cardiomyopathy (Fig. 2).

### Cardiomyocyte apoptosis

Cardiomyocyte injury and abnormal apoptosis may occur in the early stage of DCM and are directly related to myocardial hypertrophy and heart failure in DCM. Cardiomyocyte apoptosis is increased in diabetic animal models, which leads to ventricular remodeling and reduced cardiac systolic and diastolic function [23–25]. Various factors are associated with abnormal cardiomyocyte apoptosis in DCM. Studies have shown that endoplasmic reticulum (ER) stress is a key driving force of cardiomyocyte apoptosis in DCM [26–28]. In vitro and in vivo studies have revealed the beneficial effects of chlorogenic acid and hydrogen sulfide (H_2_S) against ER stress-associated apoptosis in diabetic hearts [29, 30]. Oxidative stress in the myocardial endoplasmic reticulum induced by persistent hyperglycemia can also activate caspase cascades, eventually mediating cardiomyocyte apoptosis [31–33]. SFRP2 improves diabetic cardiomyocyte injury by regulating oxidative stress and mitochondrial dynamics through the AMPK/PGC1-α pathway [34]. Natural products, including FNDC5/irisin, curcumin, and galangin, have been reported to exert cardioprotective effects by reducing oxidative stress and inhibiting myocardial apoptosis in DCM, suggesting potential therapeutic treatments for DCM [35–37]. Zhang et al. found that high glucose could activate caspase-8 and caspase-9, the key promoters of the extrinsic and intrinsic apoptosis pathways in cardiomyocytes, and subsequently activate caspase-3, the downstream executor of apoptosis, leading to neonatal rat cardiomyocyte apoptosis [38]. Allicin inhibits cardiomyocyte apoptosis by increasing the antiapoptotic protein Bcl-2 and decreasing the proapoptotic protein Fas (Fig. 2) [39].

Recent studies have shown that abnormal energy metabolism is closely related to chronic inflammation. Inflammation, which is another important causative factor of cardiomyocyte apoptosis during the progression of DCM, can not only induce the production and release of nitric oxide (NO) in cardiomyocytes but also promote the expression of proto-oncogenes, causing left ventricular diastolic dysfunction [40]. Animal models of type 1 and type 2 diabetes (T1D and T2D) exhibit systemic inflammation induced by proinflammatory cytokines and chemokines relatively early in disease progression [41]. The imbalance in blood glucose metabolism in DCM patients leads to the activation of white blood cells and the recruitment of inflammatory neutrophils, monocytes, and macrophages to the heart. Hyperglycemia also triggers the release of proinflammatory cytokines, including interleukin-1β (IL-1β), IL-6, IL-18, tumor necrosis factor-α (TNF-α) and transforming growth factor-β (1 TGF-β1), which leads to cardiomyocyte apoptosis, promoting the occurrence of diabetic cardiomyopathy [42]. The increase in ROS and NLR family pyrin domain-containing 3 (NLRP3) inflammasomes in cardiomyocytes induced by persistent high glucose can also cause inflammation, cardiomyocyte apoptosis, and myocardial fibrosis and exacerbate the development of DCM [31, 43, 44]. Evidence has shown that excessive changes in or regulation of NLRP3 inflammasome activity can promote the immunomodulatory response in DCM mice, whereas NLRP3 gene silencing can improve diabetic cardiac remodeling and impair cardiac function (Fig. 2) [45].

In addition, it is well known that intracellular Ca^2+^ is the major ion that mediates myocardial contraction. However, abnormal gene transduction of Ca^2+^-ATPase in the cardiac sarcoplasmic reticulum was found in diabetic rats, which was characterized by decreased uptake of calcium in the sarcoplasmic reticulum and a relative decrease in Na^+^-Ca^2+^ exchange in the cell membrane, which increased Ca^2+^ influx in cardiomyocytes. This impaired Ca^2+^ handling, resulting in increased action potential duration, a shortened systolic phase, and a prolonged diastolic phase in the myocardium [46–48]. Ca^2+^ overload also induced the excessive uptake of Ca^2+^ by mitochondria, increased the permeability of mitochondria, and opened the transition pore more easily, causing cardiomyocyte apoptosis [28]. Calcium signaling is also involved in the regulation of the NLRP3 inflammasome, and high levels of cytosolic Ca^2+^ further trigger NLRP3 inflammasome assembly. This effect may be due to the enhanced activation of JNK by intracellular Ca^2+^, which activates the NLRP3 inflammasome [49]. Autophagy is mainly responsible for recycling/clearing damaged organelles and cytoplasmic contents. In DCM, autophagy dysfunction impairs the fusion of autophagosomes and lysosomes in cardiomyocytes and affects the recycling and degradation of excess substances in cells. Activation of the autophagic response can prevent apoptosis and maintain normal cellular function in T1D and T2D [50]. Mitophagy, which is the highly specific elimination of dysfunctional mitochondria through the lysosome system, can also participate in DCM, suggesting an important target for delaying the progression of DCM (Fig. 2) [51, 52].

### Myocardial fibrosis

Myocardial fibrosis is one of the main histological manifestations of DCM, and increases the stiffness of the myocardium, reduces cardiac diastolic function, causes systolic dysfunction, and eventually induces sudden death. Interstitial collagen deposition was observed in cardiac sections of DCM samples, especially in the perivascular areas [53, 54]. This phenomenon has been documented in animal models of type 1 mellitus, which is induced by the administration of STZ, and type 2 diabetes, which is genetic or induced by high-fat diet feeding. At 4 months of age, db/db mice exhibit marked cardiac fibrosis. Long-term hyperglycemia and severe insulin resistance promote the proliferation of cardiac fibroblasts, which are the key cellular initiators and effectors of cardiac fibrosis, thereby increasing the production of ECM. Multiple signaling pathways are involved in myocardial fibrosis in DCM. The mechanism of the renin–angiotensin–aldosterone system (RAAS) in the progression of diabetes to heart failure is well known. Many studies have confirmed that the activation of the RAAS is closely related to myocardial hypertrophy and fibrosis in DCM patients [6, 55]. The increase in angiotensin II stimulates angiotensin receptor-1 (AT-1) to act directly on cardiomyocytes and cardiac fibroblasts, increasing collagen synthesis, decreasing collagen decomposition, causing cardiac hypertrophy and fibrosis, and resulting in reduced ventricular compliance and cardiac systolic and diastolic dysfunction (Fig. 2) [56, 57].

Matrix metalloproteinases (MMPs) and tissue inhibitors of metalloproteinases (TIMPs) are important antagonists in the regulation of ECM degradation. Studies have shown that the expression of MMP-2 is downregulated in STZ-induced diabetic mice, which is concomitant with the reduction in collagen degradation and distinct myocardial fibrosis [58]. It has also been found that activated MMP-2 can induce cardiomyocyte apoptosis through the mitochondrial apoptosis pathway [59]. In addition, high glucose stimulation affects the balance between MMPs and TIMPs, leading to an imbalance in the synthesis and degradation of ECM and collagen and promoting myocardial fibrosis. However, the specific mechanism is still unclear. Furthermore, substantial evidence has shown that the initiation of cardiac fibrosis is characterized by inflammasome complex formation in DCM [54]. The increase in the inflammatory cytokine TNF-α induced by hyperglycemia and insulin resistance can trigger cell damage and eventually lead to myocardial fibrosis in DCM patients [60]. In a rat model of DCM, inhibiting TNF-α could reduce myocardial fibrosis and improve cardiac function [61]. Nuclear factor-κ-gene binding (NF-кB), which is a transcription factor of various inflammatory factors, regulates the expression of proinflammatory, profibrotic, and hypertrophy-related genes. The excess glucose in diabetic patients promotes the generation of AGEs, which bind to specific receptors on the cell membrane, induce the release of large amounts of ROS, activate NF-кB, and then initiate the transcription of TNF-α, IL-6, and other inflammatory factors, eventually leading to vascular endothelial cell damage and smooth muscle cell proliferation and promoting cardiac fibrosis in DCM [62].

TGF-β1 is a central regulator of cardiac fibroblast proliferation and differentiation and exerts a strong profibrotic effect on other cells in the myocardium, causing and contributing to the progression of myocardial fibrosis [63]. Many factors can induce TGF-β-mediated signaling responses in diabetic hearts, such as increased levels of angiotensin II, cytokines, chemokines, integrins, and ROS, by activating local stores of TGF-β, promoting the transcription and secretion of TGF-β isoforms and inducing the synthesis and externalization of TGF-β receptors (TGFβr1 or TGFβr2) on the cell surface [64–67]. The accentuation of TGF-β signaling in the myocardial tissue of DM models causes abnormal ECM accumulation through canonical Smad-dependent pathways and non-Smad pathways. TGF-β1 activates the phosphorylation of Smad2 and Smad3 and downregulates the expression of Smad7 in models of T1D and T2D, which are associated with cardiac fibrosis [58, 68–70]. On the other hand, TGF-β1-mediated Smad-independent ERK or c-Jun amino-terminal kinase (JNK) and p38-mitogen-activated protein kinase (MAPK) signaling pathways coordinate with Smad-dependent signaling in the nucleus to promote the transcription of profibrotic markers, such as α-smooth muscle actin (α-SMA), ECM proteins (collagen I, collagen III, and fibronectin), platelet-derived growth factor (PDGF), fibroblast growth factor (FGF) and angiogenic growth factor (VEGF) [71, 72]. Strikingly, the exact mechanisms and direct evidence of TGF-β signaling pathways involved in the cardiac fibrosis of DCM are insufficient and need to be further investigated (Fig. 2).

## Therapeutic interventions of DCM

**Fig. 3:**
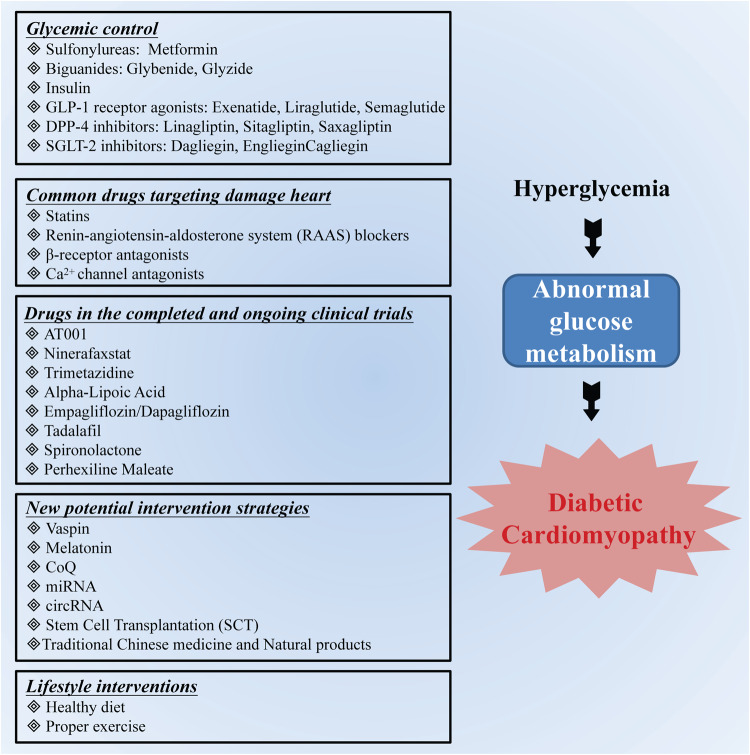
Targeted interventions for DCM.

The optimal targeted intervention strategy for DCM is to effectively control blood glucose and reduce cardiotoxicity. Common oral drugs available for glycemic control include sulfonylureas, biguanides, and insulin. Sulfonylureas stimulate islet β cells to release insulin without affecting the synthesis of patient insulin. Adversely, this class of drugs is limited to patients whose islet beta cell function remains intact and has no effect on patients whose islet beta cell function is impaired. Furthermore, long-term use of these drugs may induce a hypoglycemic response [73, 74]. Metformin treatment has a low risk of mortality in diabetic patients with heart failure compared with sulfonylurea or insulin treatment [75]. Metformin, which is a first-line antidiabetic drug, ameliorates DCM by regulating glycolipid metabolism, reducing AGE generation, inhibiting the NLRP3 inflammasome, and improving mitochondrial function, resulting in a cardioprotective effect [44, 76]. However, the latest evidence showed that metformin did not prevent the progression toward cardiac dysfunction in early DCM mice induced by a high-fat high-sucrose diet (HFHSD) [77]. Metformin treatment can impair the homing of implanted bone marrow-derived mesenchymal stem cells (BM-MSCs), reducing the efficacy of mesenchymal stem cell therapy for cardiac repair during DCM [76]. Thus, more new classes of antihyperglycemic agents have emerged. Glucagon-like peptide-1 (GLP-1) receptor agonists, such as exenatide, liraglutide, and semaglutide, can enhance myocardial sensitivity to insulin, improve the glucose uptake rate, promote myocardial energy metabolism, and inhibit cardiomyocyte apoptosis, which contributes to the treatment of DCM [78–80]. Dipeptidyl peptidase-4 (DPP-4) inhibitors, such as linagliptin, sitagliptin, and saxagliptin, can prevent cardiac dysfunction by inhibiting the Nlrp3/ASC inflammasome and modulating the JAK/STAT signaling pathway [81, 82]. It is worth noting that DPP-4 inhibitors do exert some cardioprotection in preliminary studies and initial data from phase 2 to 3 clinical trials, whereas subsequent CV outcome trials failed to show any superiority compared with the placebo in type 2 DM patients. Saxagliptin was associated with an unexpectedly increased risk of hospitalization for heart failure [83]. Sodium-glucose cotransporter type 2 (SGLT-2) inhibitors, which are another class of oral glucose-lowering agents that prevent the reabsorption of glucose, can significantly affect cardiac function in models of DCM, providing a new and promising therapeutic option for DCM [84–86]. Since insulin is not involved in this process, these inhibitors can be used at any stage of T2DM (Fig. 3).

In addition to glycemic control, a variety of drugs have been shown to have positive effects on the heart in DCM models. Statins that inhibit cholesterol synthesis have been shown to reduce the expression of vascular endothelial nitric oxide synthase, decrease the synthesis of ROS, improve left ventricular function, and inhibit myocardial fibrosis to prevent DCM [87, 88]. However, long-term clinical data showed that while these drugs reduce the occurrence of adverse cardiovascular events, lipophilic statins can increase blood glucose levels and exacerbate the severity of diabetes, which suggests that the changes in blood glucose should be monitored in patients with DCM during the clinical use of statins [89–91]. RAAS blockers can reduce blood pressure and insulin resistance and improve myocardial diastolic function in DCM [92, 93]. Although the incidence and mortality due to cardiovascular disease in diabetic patients taking angiotensin receptor blockers (ARBs) and angiotensin-converting enzyme inhibitors (ACEIs) were decreased, the effect was not obvious [94]. Spironolactone, which is an aldosterone antagonist, can inhibit the oxidative stress response in diabetic mice by enhancing the antioxidant activity of glutathione peroxidase and catalase to improve cardiac function [95, 96]. β-receptor antagonists can reduce cardiomyocyte uptake of glucose, increase the levels of oxidative stress factors (SOD1 and SOD2), and inhibit the production of oxidative stress products in DCM to modulate cardiac metabolism and improve cardiac function [97, 98]. Ca^2+^ channel antagonists can directly act on L-type Ca^2+^ channels to reduce intracellular Ca^2+^ concentrations, thereby alleviating cardiac damage in patients with DCM [99]. Long-term verapamil treatment can effectively improve myocardial hypertrophy and fibrosis by regulating of the intracellular and extracellular Ca^2+^ balance (Fig. 3) [100, 101].

Of note, there are some new and old small-molecule chemical drugs in completed and ongoing clinical trials for the treatment of DCM (Table 1). Among them, AT-001 (Caficrestat) and Ninerafaxstat (IMB-1018972) are newly-developed small-molecule chemical drugs for the treatment of DCM. AT-001 is a novel and potent oral aldose reductase inhibitor targeting AKR1B1 that is in phase 3 clinical development for the treatment of DCM (https://synapse.zhihuiya.com/clinical-progress-detail/0a5aea2aa5058d22852a258dea9d44aa). A clinical trial (NCT04365699) on the role of COVID-19 with and without treatment with AT-001 on cardiac structure and function in patients hospitalized for the management of COVID-19 infection was completed and revealed a reduction in the mortality of the AT-001 treatment group (https://synapse.zhihuiya.com/clinical-progress-detail/ed5e59202eae554e9d29a40542aea820). A clinical study evaluating the effects of Ninerafaxstat on myocardial energetics, metabolism, and functions in T2DM and obesity with HFpEF is still underway. Trimetazidine, an inhibitor of ACAA2, can improve DCM by inhibiting Nox2/TRPC3-induced oxidative stress [102], reducing the deposition of fatty acids [103], preventing fibrosis, reducing apoptosis, and enhancing autophagy [104]. The effects of trimetazidine on left ventricular function and inflammatory markers in type 2 diabetic patients were tested in a phase 2 clinical trial (https://synapse.zhihuiya.com/clinical-progress-detail/55d254aea55e524d82a2e2534e5e282e). Alpha-lipoic acid, which is a small molecule with antioxidant properties, was reported to alleviate cardiac remodeling in the diabetic heart [105], and a clinical study of the effects of alpha-lipoic acid on DCM is ongoing (https://synapse.zhihuiya.com/clinical-progress-detail/08490325eaa4e895d5a0e53324e54822). Empagliflozin and dapagliflozin, which are the initial drugs targeting SGLT2, were shown to protect cardiac function in diabetic models [106–108]. Continuous PDE5 inhibition by Tadalafil can exert beneficial effects on the cardiorenal complications of T2DM [109] (https://synapse.zhihuiya.com/clinical-progress-detail/04e25ea32d0d3a889adae2a208aae855). The antifibrotic effects of spironolactone on hearts in type 2 diabetes mellitus patients were detected by noninvasive cardiac imaging in a clinical trial (https://synapse.zhihuiya.com/clinical-progress-detail/9a22aa02522ea9d092ea289500d5ed5a). The mechanisms responsible for abnormal cardiac energetic metabolism and the effects of perhexiline, which improves metabolic impairment in diabetic patients before the development of heart failure, were explored and assessed in a clinical study (https://synapse.zhihuiya.com/clinical-progress-detail/222ae22992aeaa3a0ee394ae852a24d2).

**Table 1:** Drugs in completed and ongoing DCM-related clinical trials.

Name	Target	Clinical phase	ClinicalTrials.gov ID	Status
AT001	AKR1B1	Phase 3	NCT04083339	Active, not recruiting
Ninerafaxstat	-	Phase 2	NCT04826159	Recruiting
Trimetazidine	ACAA2	Phase 2	NCT05556005	Recruiting
Alpha-Lipoic Acid	-	N/A	NCT04141475	Recruiting
Empagliflozin	SGLT2	Phase 1	ChiCTR2000038485	Recruiting
Dapagliflozin	SGLT2	N/A	NCT04591639	Recruiting
Tadalafil	PDE5A	Phase 4	NCT01803828	Completed
Spironolactone	MR	N/A	ACTRN12609000789268	Completed
Perhexiline maleate	CPT2, CPT1A	Phase 1/2	NCT00628056	Unknown

Over the years, researchers have discovered some new intervention strategies, such as vaspin, melatonin, CoQ, miRNA, circRNA, and stem cell transplantation (SCT), which have certain protective effects on the heart in DCM [110–114]. Moreover, traditional Chinese medicine and some natural products, such as ginsenoside, esveratrol, berberine, curcumin, epigallocatechin gallate, flavonoid, ginkgo biloba extract, astragalus polysaccharide, have been confirmed to exert antioxidant, anti-inflammatory, antiapoptotic, and antidiabetic effects, playing important roles in the regulation of cardiac function in models of DCM [50, 115]. The Chinese herbal preparations Fufang Zhenzhu Tiao Zhi (FTZ), Erzhi Pill (EZP) and Si-Miao-Yong-An decoction (SMYA) can alleviate DCM by reducing oxidative stress and NLRP3 inflammasome activation, inhibiting apoptosis and improving energy and glucolipid metabolism through different signaling pathways [116–118]. Lycium chinense leaf extract (LCME) protects against DCM by inhibiting oxidative stress, inflammation, apoptosis, and fibrosis [119]. *Panax notoginseng* saponin can improve diabetic cardiomyopathy by reducing lipotoxicity, inhibiting oxidative stress and enhancing mitochondrial function [120]. Notoginsenoside R1 (NGR1) exerts cardioprotective effects against DCM by suppressing cardiac fibrosis and hypertrophy [121]. This evidence suggests that Chinese herbal prescriptions and natural products can provide new sources of drug candidates to protect against DCM (Fig. 3). Finally, lifestyle interventions are essential. It is well known that a healthy diet and proper exercise are beneficial for improving glycemic and weight control and promoting insulin sensitivity to reduce the burden on the heart in DCM (Fig. 3) [43, 122].

## Conclusion

DCM, which is one of the main complications of diabetes, is considered to be a lesion in myocardial structure and function caused by diabetes without atherosclerosis in the coronary artery or hypertension. Due to the atypical clinical manifestations of DCM, current clinical diagnosis of this disease is limited and is mainly based on the comprehensive evaluation of the patient’s medical history, symptoms, echocardiography, magnetic resonance imaging, and some emerging biomarkers, such as serum HbA1c and cTnI levels. Unexpectedly, the sensitivity and specificity of these diagnostic methods are not sufficient. Moreover, the pathogenesis of DCM is complex, diverse, and not fully understood. Abnormal glucose and lipid metabolism leads to insulin resistance, mitochondrial dysfunction, ERS, inflammation, calcium stability imbalance, autophagy, cardiomyocyte apoptosis, and myocardial fibrosis. These factors interact with each other, further exacerbating energy metabolism disorders and inflammatory reactions. Herein, we introduced the action mechanisms of common drugs and targeted intervention strategies used for the treatment of DCM and summarized the advantages and limitations. Although the current intervention strategies can alleviate the symptoms of DCM to a certain extent, they cannot effectively reverse myocardial injury. Thus, there is still a lack of specific therapies. It is urgent to further explore and understand the pathophysiological mechanism of DCM to provide a new direction for improving prognostic methods and developing new therapeutic targets and drugs with good efficacy and fewer side effects.
